# The Impact of Carbon Trading Pilot Policy on Carbon Neutrality: Empirical Evidence from Chinese Cities

**DOI:** 10.3390/ijerph20054537

**Published:** 2023-03-03

**Authors:** Ke Zhang, Jing Qian, Zhenhua Zhang, Shijiao Fang

**Affiliations:** 1 School of Economics, Zhongnan University of Economics and Law, Wuhan 430073, China; 2 Institute of Green Finance, Lanzhou University, Lanzhou 730000, China

**Keywords:** carbon trading pilot, carbon neutrality, net carbon sink

## Abstract

As one of a number of crucial policies for achieving the goal of “double carbon”, it is crucial to investigate the “carbon neutral” effect of the carbon trading market (CTM) in the pilot phase, which is an essential reference for the development of a future CTM. Based on panel data of 283 cities in China in the period from 2006 to 2017, this paper examines the impact of the Carbon Trading Pilot Policy (CTPP) on the achievement of the “carbon neutrality” target. The study shows that the CTPP market can promote an increase in regional net carbon sinks and further accelerate the achievement of the “carbon neutrality” goal. The findings of the study remain valid after a series of robustness tests. The mechanism analysis finds that the CTPP can help achieve the carbon neutrality target through three mechanisms: the effect on concern for the environment, the effect on urban governance, and the effect on energy production and consumption. Further analysis reveals that enterprises’ willingness and productive behavior, as well as the internal elements of the market, have a positive moderating effect on the achievement of the carbon neutrality target. In addition, there is heterogeneity among regions with different technological endowments, CTPP regions, and regions with different shares of state-owned assets in the CTM. This paper provides important practical references and empirical evidence that can help China to better achieve the “carbon neutrality” target.

## 1. Introduction

With the intensification of global warming and air pollution, the natural ecological environment on which people depend has been significantly damaged, and countries all over the world are actively exploring ways and means to deal with the problem of climate change. As a practical initiative derived from the new institutional economics and property right theory, the carbon emission trading market (hereafter referred to as the “carbon trading market” (CTM)) is generally regarded as an effective means of reducing global greenhouse gas emissions and combating climate change [1,2]. As a responsible global power, China has been actively implementing a Carbon Trading Pilot Policy (CTPP), promoting the development of a national CTM, and integrating this into the development of a national ecological civilization [3].

The CTPP means that China has pioneered regional carbon trading markets (CTM) in some cities, and then a national CTM will be established at a comprehensive level. The CTPP is a market-incentive-based system of environmental regulation by economic means, which aims to use market mechanisms to reduce carbon emissions. The way the CTPP works is that the government first assesses the industry nature of the enterprise, its historical carbon emission status, and the total amount of carbon emission reduction in the region and then determines the initial amount of carbon emission rights the enterprise has. In the case that a company’s carbon emissions exceed its initial allowance, it will need to purchase carbon emissions in the CTM, and if it does not exceed its initial allowance, then the company’s surplus carbon emissions can be sold in the CTM. In this way, with the market mechanism, the CTPP not only enables the total carbon emissions of the whole region to be controlled but also enables the regional economy and technological innovation to be further developed. Therefore, compared to other command-and-control environmental projects in China, such as Low carbon city and the recent Ecological and Environmental Monitoring Measures for Ecological Protection Red Line (Trial), the CTPP is a win-win environmental policy that achieves economic and environmental benefits through the market mechanism.

With the continuous development of the CTM, the first compliance period of the China CTM has also ended. In the first compliance period, China’s carbon market included 2162 power generation enterprises and covered greenhouse gas emissions amounting to 4.5 billion tons of carbon dioxide. The cumulative transaction volume of the carbon emission quota is 179 million tons, with a transaction value of RMB 7.661 billion, and the average transaction price of the carbon quota is RMB 42.85 per ton. It can be seen that the results of China’s first CTM compliance period are relatively remarkable, and the future development of the CTM is in good shape.

The Chinese government’s 2022 Work Report further emphasizes the orderly promotion of carbon peaking and carbon-neutral work, and one critical task in this area is to accelerate the development of the CTM. China is expected to build the world’s largest CTM covering greenhouse gas emissions. This will also be an essential step in China’s response to global climate change [2].

During the decade from 2011, when the plan for developing the CTM was proposed, to 2021, when the online trading of the national CTM was launched, the CTM has contributed significantly to the realization of China’s “double carbon target (On 22 September 2020, President Xi Jinping solemnly announced at the 75th session of the United Nations General Assembly that “China will strive to reach peak CO_2_ emissions by 2030 and work towards achieving carbon neutrality by 2060”)”, and its carbon emission reduction effect has been widely recognized by the academic community [1,2,4,5]. However, it is worth noting that although the “dual carbon targets” have the same origin, there is a fundamental difference between the two mechanisms for reducing the accumulation of greenhouse gases in the atmosphere. Specifically, the ultimate goal of the “dual carbon goals” is to reduce the accumulation of greenhouse gases in the atmosphere. The solutions for doing so can be approached from two perspectives: First, reducing carbon sources, and so reducing greenhouse gas emissions; Second, increasing carbon sinks, and so increasing the absorption of greenhouse gases, especially carbon dioxide [6,7,8]. Therefore, in regard to the “carbon peaking” target, as long as the total or marginal amount of carbon sources is decreasing, achievement of the “carbon peaking” target can be promoted. In contrast, in regard to the “carbon neutral” target, the goal of “carbon neutrality” can be achieved not only by reducing carbon sources but also by increasing the total or marginal amount of carbon sinks, achieving an increase in net carbon sinks. In other words, the core way of achieving the goal of “carbon neutrality” is to increase the number of carbon sinks over the number of carbon sources [8,9,10]. The positive impact of the current CTM on the “double carbon target” is only focused on the “carbon peak” target. Can the CTM effectively contribute to the “carbon neutral” target? In other words, can the CTM effectively contribute to achieving the “carbon neutral” goal by increasing net carbon sinks? There is still little in-depth analysis of this point in the literature. The answer to this question is not only a test of the effectiveness of the current CTPP implementation but also an essential reference for the development of the national CTM and for the future direction of the deepening of the “double carbon” goal. At the same time, the answer to this question can also further promote previous research. Therefore, this paper presents a comprehensive assessment and analysis of the carbon neutrality effect of the CTPP and its influence mechanisms. The paper concludes that the CTPP can significantly contribute to increasing the net carbon sink and can further promote the achievement of the “carbon neutrality” goal. In addition, this paper also finds that the CTPP can help achieve the “carbon neutrality” target through three mechanisms: the effect on concern for the environment, the effect on urban governance, and the effect on energy production and consumption.

The main contribution of this study lies in three areas: Firstly, unlike previous studies, this paper focuses on the net carbon sink and constructs a multi-period DID to investigate the impact of the CTPP on the net carbon sink, which provides more specific recommendations for achieving the goal of “carbon neutrality”. Secondly, to understand the mechanisms by which the CTPP promotes the achievement of the goal of carbon neutrality, this paper discusses three aspects—the government’s concern for the environment, urban governance, and energy production and consumption, which are helpful in regard to improving the effectiveness of the practice in reality. Finally, as China is the most carbon-emitting country in the world, a comprehensive assessment and analysis of the “carbon neutral” effect of the pilot CTM and its impact mechanisms will not only provide an essential practical reference and empirical basis for China’s efforts to better achieve the “double carbon” target but will also provide an essential reference for the world’s carbon control and development efforts. It can also provide solutions with Chinese characteristics, for controlling the world’s carbon stock and mitigating climate change.

The rest of the paper is structured as follows: the second section presents the literature review; the third section presents the theoretical analysis and research hypothesis; the fourth section presents the research design; the fifth section presents the empirical analysis; the sixth section presents the research’s limitations; and, finally, the seventh section presents the conclusion and recommendations.

## 2. Literature Review

In the current research exploring the relationship between the CTPP and carbon neutrality, the literature mainly focuses on the model derivation of the emission reduction effect of the CTM, the evidence test of the emission reduction effect of the CTM, and the economic effect brought about by the CTM. Firstly, in terms of modeling the emission reduction effect of the CTM, the existing literature mainly simulates China’s CTM through the CGE model [11], the multi-agent model [12], and the multi-regional general equilibrium model [13], and the findings using these models show that the CTM can bring a significant emission reduction effect and a significant economic effect. Secondly, in terms of evidence testing of the emission reduction effect of the CTM, most of the existing literature has been conducted at the national, provincial, and enterprise levels using the double difference method and the synthetic control method [1,3,4,14,15,16,17], and the findings using these methods also indicate that the CTM can play a significant role in curbing carbon emissions and other pollutant emissions through mechanisms such as technological innovation, industrial restructuring, and energy restructuring. Of course, only a small number of papers have examined the evidence of the emission reduction effect of the CTM at the city level [2,5]. In addition, since an essential consequence of carbon emissions is increased air pollution and an increased public health burden, some studies have also explored the impact on air pollution and public health of the policy on the atmospheric environment and thus have further explored the impact on human health [18,19]. Finally, regarding the economic effect brought about by the CTM, the existing literature agrees that the CTM can play a significant role in promoting economic growth and a green economy through mechanisms such as the expansion of the market scale, technological improvements, different emission right allocation methods and the synergy of related environmental policies [14,16,20,21,22].

In addition, in studies related to reducing the amount of carbon dioxide in the air, the current literature focuses on reducing carbon sources and increasing carbon sequestration. In terms of carbon source reduction, scholars have investigated the factors relating to carbon source reduction from various perspectives, such as the Air Pollution Prevention and Control Action Plan [23], new urbanization [24], energy use [25], trade linkages [26], clean production [27], urban transportation [28], industrial emissions [29], and green finance [30], and have achieved fruitful research results. In terms of carbon sequestration, existing studies have mainly measured the carbon sequestration capacity of forests [31], grasslands [32], crops [7], and vegetation [33], and have indicated that nature itself has great carbon sequestration potential and carbon sink value, which should be fully utilized and protected. Regarding the net carbon sink, which is closely related to the “carbon neutral” target, the existing literature focuses only on analyzing the varying spatial and temporal characteristics of the net carbon sink of conservation tillage [34], farmland ecosystems [35], agriculture [36], plantations [37], and its driving force. All of these studies consistently show that the net carbon sink effect is of great practical significance for mitigating global warming and achieving the national “double carbon” goal.

In summary, the existing studies provide a good reference for this paper, but the following shortcomings can be highlight. First, the current literature mainly focuses on assessing the CTPP at the provincial level and tends to group Shenzhen and Guangdong into one provincial level and as a unified treatment group. Although there are a few papers that have tested and evaluated the CTM at the city level, due to data limitations, most of their city-level carbon emission data are estimated based on the energy volumes of local statistical yearbooks, which introduces significant measurement errors into the carbon emission data of prefecture-level cities, thus reducing the accuracy of research conclusions and increasing the endogeneity of the models used. Second, in the current research on the relationship between the CTPP and total CO_2_ emissions, most studies focus on the emission reduction effect of the CTPP but tend to ignore the net carbon sequestration effect of the CTPP, even though considering whether the CTPP can promote an increase in the net carbon sink goes to the core of the “carbon neutrality” goal [8,9]. Therefore, this paper differs from existing studies in the following ways. Firstly, this paper takes a sample of cities as the research object, differentiates the CTM in Guangdong and in Shenzhen, and applies a multi-period double difference method to evaluate the impact of the CTPP (seven official pilot areas for carbon trading and one voluntary pilot areas for carbon trading) on the “carbon neutrality” target. This paper will enrich existing research results through its overall evaluation and mechanism analysis. At the same time, this paper uses the more authoritative and accurate regional carbon emission data and regional carbon sequestration data [33] to conduct the corresponding empirical tests, which helps to increase the credibility and accuracy of the research findings. This will not only help enrich the current research on CTM assessment but also provide new thinking and theoretical support for the CTM in regard to promoting the goal of “carbon neutrality”.

## 3. Theoretical Mechanisms and Research Hypotheses

As a typical environmental “public good”, carbon emissions are challenging to solve the external environmental diseconomies arising from economic activities through spontaneous market mechanisms [38]. The CTM treats carbon dioxide emission rights as a commodity. Trading emission rights has the advantage of controlling carbon emissions in total. In the face of the double pressure of international emission reduction commitments and the continuous downward trend of domestic environmental conditions, China has moved from the pilot phase to the full-scale expansion of the CTM. It has been established that the carbon emission reduction effect of the CTM has been achieved in China and that the CTM is an effective means of achieving low-carbon development [4]. The goal of “carbon neutrality” can be achieved not only by reducing carbon sources but also by further increasing the total or marginal amount of carbon sinks, as long as the net carbon sink (carbon sinks are more excellent than carbon sources) is increased [8,9,10], then it can further accelerate the achievement of carbon neutrality. The effectiveness of the CTM in reducing emissions is now widely recognized [1,2,4,5]. Therefore, if carbon sinks remain unchanged, or at least do not decrease, this means the CTPP has a significant positive effect on the net carbon sinks, which can further promote the achievement of the “carbon neutrality” goal. Therefore, this paper proposes Hypothesis 1:

**H1.**  *The CTPP can promote an increase in the net carbon sink and accelerate the achievement of the “carbon neutral” goal.*

### 3.1. Effect on Concern for the Environment

The CTM in China has brought huge economic benefits since the start of the CTPP [14,16,20,21]. Since China’s CTM follows the principle of “total control quota”, a reduction in the total amount of carbon emissions in each region will lead to an increase in carbon allowances as compared to the original total amount of carbon emissions [39], which will provide a significant boost to the economy [40]. Under the pressure of economic growth targets, local governments are bound to pay close attention to the carbon emission situation and the economic situation in the CTM, thus increasing concern for the environment [41]. As the government pays more attention to the environment, the awareness of environmental protection and sustainable development in the region will be strengthened, and the sense of responsibility for environmental protection of each individual in the economy will be further increased so that they will practice a simple, moderate, green, low-carbon, civilized and healthy life, and similar production patterns in various aspects, and thus will effectively reduce their resource consumption and energy carbon emissions [42], which will eventually lead to an increase in the net carbon sink. This will ultimately lead to an increase in the net carbon sinks and accelerate the achievement of the goal of “carbon neutrality”. Therefore, this paper proposes Hypothesis 2:

**H2.**  *The CTPP can further promote an increase in the net carbon sink and accelerate the achievement of the “carbon neutrality” goal by increasing the government’s concern for the environment.*

### 3.2. Effect on Urban Governance

The aim of the CTPP is to achieve a win-win situation for both the environment and the economy. As a firm implementer, administrator, and public service provider of this policy, the governments of the pilot areas for carbon trading should take the lead in not only maintaining the operation of the carbon market but also implementing and exercising responsibility for, and fulfilling the obligations of, the “visible hand” [31], to earmark part of the revenues from the carbon market for “green spending” related to environmental protection [43] and to achieve twin progress on ecological and environmental protection and economic development [44]. One of the most important aspects is the strengthening of urban pollution control. By strengthening urban pollution control, the government can not only protect soil vegetation, restore its ecological capacity, enhance its ability to absorb pollutants, and strengthen the carbon sequestration capacity of ecosystems but also significantly increase the “carbon absorption” capacity of soil and plants, ensure the livability of the ecological environment in the region, improve the comfort and quality of life of residents, optimize the regional carbon sink pattern [42,44], and further increase the net carbon sink. At the same time, it can also recycle domestic wastewater, industrial waste, and other domestic production waste, realize a circular economy, accumulate green capital, improve cities’ ecological environment, and further reduce carbon emissions. Therefore, this paper proposes Hypothesis 3:

**H3.**  *The CTPP can further promote the increase in the net carbon sink by increasing the pollution control in cities and accelerating the achievement of the “carbon neutrality” goal.*

### 3.3. Effect on Energy Production and Consumption

China’s primary source of carbon emissions is energy emissions generated from economic production activities. Therefore, in addition to the emission reduction effect brought about by the CTM, the reduction of carbon stock in the air can be considered at two levels: on the production side and on the energy consumption side. First, in regard to the energy production side, under the constraint of carbon quota, the carbon trading system can stimulate energy-intensive enterprises to develop and invest in renewable energy [4,41], thus increasing the production of renewable energy in the region and alleviating the reliance on non-renewable resources, such as coal, and ultimately reducing the carbon source. In addition, coal development is often accompanied by reducing the available vegetation and high-quality ecological area, thereby reducing the regional carbon sink. At the same time, an increase in renewable energy production will further inhibit the destruction of the local natural environment, thus further increasing the carbon sink. Second, China’s energy endowment has determined the irreplaceable role of coal in the country’s early industrialization [45]. Under the CTM quota constraint, high coal-consuming enterprises often need to purchase carbon emissions rights above their quotas in the CTM, based on the need to expand their production scale, which inevitably leads to an increase in the cost of the enterprises, and thus the cost caused by the purchase of carbon emission rights is included in the production cost when these enterprises conduct a cost-benefit analysis. This poses a serious challenge to the market competitiveness of these enterprises [1]. Therefore, under the pressure of the CTM, high coal-consuming enterprises will further reduce the intensity of their coal consumption and energy consumption [4], lowering the regional carbon source. In turn, the reduction in coal consumption will further increase the region’s carbon sink. Therefore, this paper proposes Hypothesis 4:

**H4.**  *The CTPP can further promote an increase in the net carbon sinks and accelerate the achievement of the “carbon neutrality” goal by increasing the consumption of renewable energy and reducing the consumption of coal and the intensity of energy.*

## 4. Study Design

### 4.1. Model Setting

Since the eight pilot regions (seven official pilot areas for carbon trading and one voluntary pilot area for carbon trading) (Beijing, Tianjin, Fujian, Guangdong, Shanghai, Shenzhen, Hubei, and Chongqing are the official pilot areas for carbon trading; Fujian is the voluntary pilot areas for carbon trading) did not implement carbon markets at the same time, this paper adopts a multi-period DID to test the impact of carbon markets on regional “carbon neutrality” targets, following the approach of Wu et al. (2021) [2]. The core idea is to select an experimental group with a carbon market and a control group without a carbon market and then to consider the difference between the average change in the dependent variables before and after the implementation of the policy in the experimental group and the average change in the dependent variables before and after the implementation of the policy in the control group, which is considered the actual effect of the policy implementation on the experimental group. With all other factors being equal, the multi-period DID can test whether there is a significant difference in the net carbon sinks between the pilot and non-pilot regions before and after the pilot regional CTM. The specific model is as follows:

(1) $$Y_{it}=\beta_{0}+\beta_{1}DID_{it}+\beta_{2}\;\mathrm{contro}l_{it}+cs_{i}+year_{t}+qy_{rt}+\varepsilon_{it}$$

where *Y_it_* is the dependent variable, which is the “carbon neutral” target and is characterized by the net regional carbon sink; $DID_{it}$ is the core independent variable, which is expressed by *treatment_i_ × post_it_* (*DID_it_ = treatment_i_ × post_it_. treatment_i_* represents whether it is a treatment group, and *post_it_* represents when the policy was implemented); *control_it_* is the corresponding set of control variables; $cs_{i}$ is city-fixed; $year_{t}$ is time-fixed;

*qy_rt_* denotes regional time-varying factors controlling for variation with region (In this paper, referring to Wu et al. (2021), Chinese cities are divided into four major regions by geographical location: east, northeast, central, and west) and over time; $\varepsilon_{it}$ is the random error term. In addition, *treatment_i_* is defined as follows: when the city term is a prefecture-level city in the eight pilot regions, *treatment_i_* is recorded as 1; otherwise, it is 0. *post_it_* is defined as follows: when the city term is a prefecture-level city under Beijing, Tianjin, Shanghai, Shenzhen, and Guangdong Province (except Shenzhen) and the time variable is ≥2013, or the city item is Chongqing and Hubei subordinate prefecture-level cities and the time variable is ≥2014, or the city term is a prefecture-level city in Fujian Province and the time variable is ≥2016, the *post_it_* is recorded as 1; otherwise, it is 0. This paper performs standard error clustering at the city level to solve the potential bias problem caused by serial correlation and heteroskedasticity. If the CTM has a significant impact on the “carbon neutrality” target, it significantly increases the regional net carbon sink, and thus *β*_1_ should be significantly positive.

### 4.2. Sample Selection and Data Description

#### 4.2.1. Dependent Variables and Core Independent Variables

The focus of this paper is on whether the CTPP can contribute to the goal of “carbon neutrality”, which can be achieved not only by reducing carbon sources but also by further increasing the total or marginal amount of carbon sinks, thus achieving an increase in the net carbon sink. In other words, the core pathway to achieving the goal of “carbon neutrality” lies in carbon sinks exceeding carbon sources [8,9,10]. Therefore, given the limitations in respect of data availability, the dependent variable in this paper is the difference between the carbon sequestration and carbon emissions of each prefecture-level city, the net carbon sink (Ncs), and the core independent variable in this paper is the multi-period DID variable of the CTPP described above.

#### 4.2.2. Control Variables

Drawing on relevant studies [2,46], given that regional economic variables are closely related to carbon emissions, economic variables that can influence carbon emissions at the regional level are further selected to form a set of control variables. Specifically, the logarithm of GDP per capita and the quadratic term of GDP per capita (Rgdp, Rgdp2), calculated at constant 2006 prices; industrial structure(Is), expressed as the share of tertiary industry; economic structure (Es), expressed as the share of retail sales of social goods; degree of openness to the outside world (Dotow), expressed as the share of actual use of foreign capital in the current year; population density (Pd), expressed as the area of the city’s population in the region; marketization index (Mi), expressed as the Fan Gang marketization index; the number of industrial enterprises (Nie), expressed as the logarithm of the number of industrial enterprises; the degree of fiscal dependence (Dfd), expressed as the proportion of general public budget revenue; the density of urban private and individual employees(Dupie), expressed as the proportion of the number of private and individual employees. The descriptive statistics of specific variables are shown in Table 1.

#### 4.2.3. Data Description

First, this paper uses a panel of 283 Chinese cities in the period from 2006 to 2017 to examine the impact of carbon market pilots on achieving the “carbon neutrality” goal. The reason for choosing 2017 as the cut-off point is as follows: First, the national CTM was officially launched at the end of 2017, and there are no more CTPPs in each region. The purpose of this paper is to conduct a comprehensive assessment of the “carbon neutral” effect of the CTM during the pilot period to investigate whether the CTM is effective in achieving the “carbon neutral” objective during the pilot period, on the one hand, and to use the results of the pilot as a basis for helping China to better achieve the “dual carbon neutral” objective, on the other hand. At the same time, we aim to provide essential practical references and experiences to help China to better achieve the “double carbon” goal. Second, considering the limitations of data availability, completeness, and scientific aspect, the data used in this paper only go up to 2017. On the one hand, this paper adopts the more authoritative and accurate regional carbon emission data and regional carbon sequestration data gathered so far [33] for the corresponding empirical test. Chen et al. (2020) [33] used the particle swarm optimization-back propagation (PSO-BP) algorithm to match and unify the DMSP/OLS and NPP/VIIRS satellite image data and obtained probably the best fit so far, with the inverse carbon emissions of 2735 counties in China from 1997 to 2017 using the highly correlated properties of nighttime lighting data and human activities. This paper also follows the practice of Wu et al.(2021) [2]. On the basis of both data [2,33], the corresponding prefecture-level data can be obtained by summing up the county data. On the other hand, in terms of carbon emission data, in most of the existing studies using city-level carbon emissions data, the date is estimated based on the energy volume of local statistical yearbooks, which leads to significant measurement errors in the actual carbon emission data of the prefecture-level cities, thus reducing the accuracy of the study conclusions. At the same time, in terms of natural carbon sequestration data, existing studies have shown that oceans, forests, soils, vegetation, and crops can sequester carbon. Ideally, the carbon sequestered by these elements should be fully measured and summed up to obtain the complete amount of natural carbon sequestration. However, due to the completeness of the actual data and the uniformity of the measurement caliber, this paper can only select the most complete and scientific data among the existing data, which can match the carbon emission data with the same measurement caliber and accuracy [33]. Third, the time cut-off point for the CTPP has been selected as 2017 [1,2,16,17,21]. In summary, this paper fixes the time cutoff as 2017. In addition, the marketization index is obtained from Fan Gang’s “China Marketization Index by Province Report”. Other data are obtained from the China City Statistical Yearbook, the China Regional Statistical Yearbook, the China Energy Statistical Yearbook, the China Industrial Economy Website, and the China Industrial Statistical Yearbook.

## 5. Empirical Analysis

### 5.1. Baseline Regression Results

This paper first explores the impact of the CTM on the net carbon sink. The specific tests are shown in Table 2.

Table 2 presents the baseline regression results for Model 1 (Equation (1)). Among them, column (1) does not add control variables, and columns (2)–(6) gradually add time-fixed, city-fixed, and region-year interaction fixed effects on top of the added control variables, respectively. All columns are also clustered with standard errors at the city level. As shown in Table 2, the coefficients of DID are significantly larger than 0. Therefore, it is shown that the CTPP can significantly contribute to increasing the net carbon sink in the region and can accelerate the achievement of the “carbon neutrality” goal. Hypothesis 1 is proved.

### 5.2. Robustness Test

#### 5.2.1. Parallel Trend Test

An important prerequisite for the results of the DID to be considered consistent is that the experimental and control groups before policy implementation conform to the parallel trend hypothesis, meaning that without the intervention of the carbon emission trading system, the dependent variables maintain relatively stable trends in both the experimental and control groups [17]. Therefore, to ensure the reliability of the results, this paper refers to Wu et al.(2021) [2] and constructs the interaction term between the year dummy variables and the corresponding policy dummy variables for the five years before implementation, in the year of implementation, and in the four years after the implementation of the CTPP for the parallel trend test, as follows:

(2) $$\begin{array}{c} Y_{it}=\beta_{0}+\overset{5}{\underset{s=1}{\sum }}\beta_{{\mathrm{preses}}_{-s}}D_{\mathrm{pre}\_s\;}+\beta_{\mathrm{current}\;}D_{\mathrm{current}\;}+\overset{4}{\underset{s=1}{\sum }}\beta_{{\mathrm{post}\_s}_{}}D_{\mathrm{post}\_s\;}\\ +\beta_{2}{\;\mathrm{control}\;}_{it}+\eta_{i}+\gamma_{t}+\delta_{rt}+\varepsilon_{it} \end{array}$$

**Figure 1:**
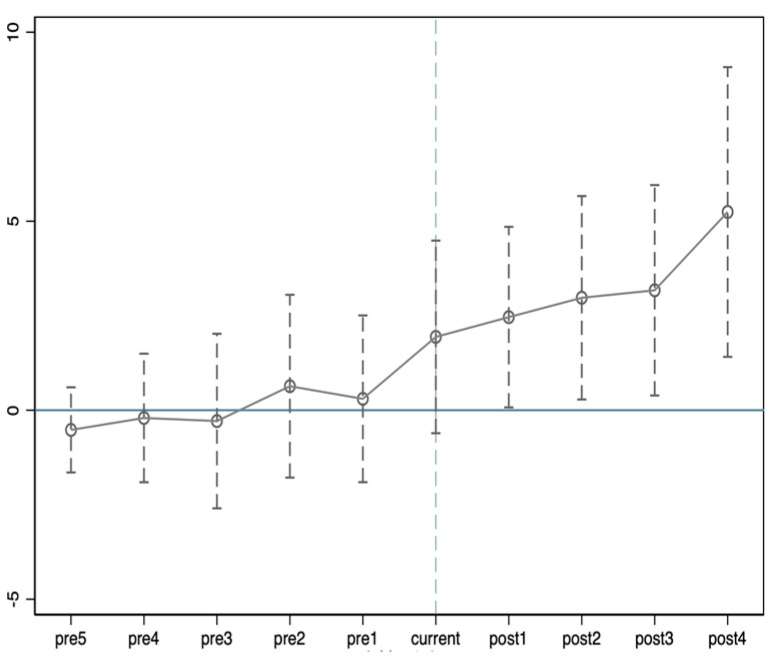
Parallel trend test.

Among them, *D_pre_s_*, *D_current_*, and *D_post_* denote the interaction terms between the year dummy variables and the corresponding policy dummy variables before implementation, in the year of implementation, and after implementation, and the other symbolic meanings are the same as in Equation (1). The specific test results are shown in Table 3 and Figure 1.

Table 3 reports the parallel trend test of the net carbon sink before and after the CTPP. It can be seen that the coefficients before the CTPP $\beta_{{\mathrm{preses}\_s}_{}}$ are insignificant and pass the significance test, which indicates that the trend of the net carbon sinks of the treatment and control groups satisfies the parallel trend hypothesis. In addition, Figure 1 also shows no significant difference between the net carbon sinks of the treatment and control groups before the implementation of the CTPP. Hence, the parallel trend hypothesis is satisfied.

#### 5.2.2. Placebo Test

**Figure 2:**
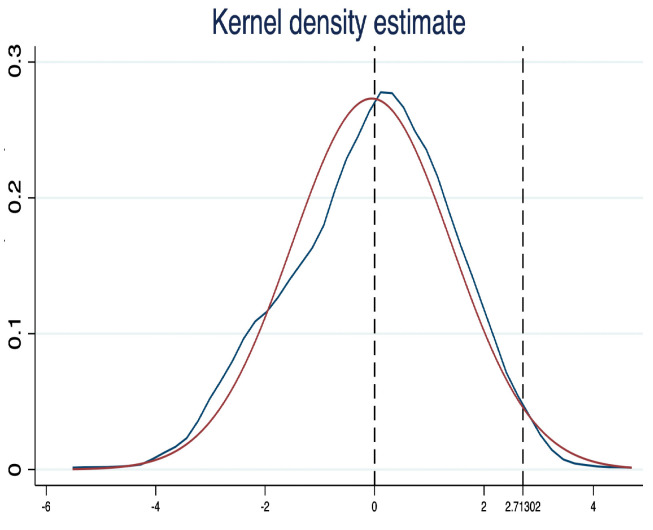
Placebo test. Note: kernel = epanechnikov, bandwidth = 0.3793.

Bertrand et al.(2004) [47] argue that when performing DID analysis over long time spans, there may be a problem of standard error bias caused by serial correlation, thus making the test overdetermined and rejecting the original hypothesis. To this end, to enhance the robustness of the benchmark regressions in Table 2, this paper refers to existing studies [2,48,49] and further employs a placebo test using a nonparametric substitution test. Specifically, unduplicated random sampling is conducted for all provincial regions and policy times. Eight provincial regions and the corresponding random policy time points of each provincial region are randomly selected each time. The cities corresponding to the eight selected provincial regions are taken as virtual treatment groups. The remaining cities are taken as virtual control groups and repeated 500 times to obtain the estimated coefficients of DID regressions for 500 virtual treatment groups and virtual policy time interactions. If the CTM has a significant net carbon sequestration effect on the pilot regions, then the DID estimate coefficients in Table 2 (2.713) should be located in the distribution’s low tail (right truncated tail) of the coefficients of the non-reference substitution test. The specific test results are shown in Figure 2. As indicated in Figure 2, it can be seen that the baseline regression results passed the placebo test.

#### 5.2.3. Other Robustness Tests: PSM-DID and Excluding the Effects of Other Relevant Policies in the Same Period

To further confirm the robustness of the baseline regression results, the DID test is conducted using the PSM-DID method, which uses the control variables in Equation (1) as covariates to match the propensity scores year by year, retaining the sample points that are within the standard range of values in each matching year, and conducting the DID test only for those samples that are within the standard range of values [2]. In addition, the paper further excludes the effects of other important policies for robustness testing. The results are shown in Table 4.

The results in Table 4 indicate that the coefficient of PSM-DID is significantly positive regardless of the matching method used. In addition, the coefficient of DID is also significantly positive after excluding other related policies in the same period. Therefore, the baseline regression in this paper is robust.

#### 5.2.4. Other Robustness Tests: Excluding the Effect of Special Samples and Replacing the Dependent Variables

To further confirm the robustness and credibility of the baseline regression results, this paper also conducts robustness tests by excluding the effects of unique samples and replacing the dependent variables. In terms of the robustness test by excluding the effects of unique samples, this paper attempts to exclude three types of special samples one at a time for the robustness test. First, this paper attempts to exclude all municipalities in the pilot cities, in addition to their greater economic strength, because the municipalities implement other strict energy efficiency and emission reduction policies, which may interfere with the identification of the policy effects of the CTPP [2]. Second, this paper tries to exclude only the Chongqing sample because Chongqing is located in the west, and its peculiar regional economic development characteristics may also affect the results of the benchmark regression. Third, this paper attempts to exclude only the Fujian and Sichuan samples, because both Fujian and Sichuan launched the CTM at the end of 2016, close to the full launch in 2017, so their exclusion can be further tested for robustness. In terms of the robustness test by replacing the dependent variables, this paper takes the logarithm of the value of carbon sequestration in the original data and the logarithm of the value of carbon emissions and takes the difference to get the new net carbon sink value for the robustness test. At the same time, considering that air pollutants and carbon emissions have the same root and source, this paper also further uses the concentration of PM_2.5_ in the atmosphere instead of the original net carbon sink for the robustness test. The specific test results are shown in Table 5.

The results in Table 5 show that the regression coefficient of DID remains significantly positive after excluding the effect of the special sample. Similarly, the regression coefficient of DID is significantly positive after replacing the measures of the dependent variables and significantly negative after replacing the meanings of the dependent variables. Therefore, the baseline regression in this paper is robust.

In addition, this paper further analyzes “Carbon emissions” and “Carbon sink”, shown in Table 6. According to Table 6, the CTPP has a significant suppressive effect on “carbon emissions” and a positive, albeit non-significant, effect on “carbon sink”. This paper argues that carbon sink by terrestrial vegetation may also be influenced by several uncertainties, such as extreme climate and temperature anomalies [8]. China has experienced frequent extreme weather and natural disasters from 2006 to 2017, such as the extremely high temperature and drought in 2006, the Wenchuan earthquake and the great snowstorm in 2008, and the floods and hailstorms in 2015. Therefore, it is possible that the positive but insignificant effect of the CTPP on “Carbon sink” is due to the influence of extreme weather and temperature anomalies. Next, this paper intends to explore other variables that reflect carbon sink by terrestrial vegetation and other carbon sink variables to determine whether the CTPP also has no significant effect on any of these variables. This paper has studied and referred to other scholars’ methods of assessing and measuring carbon sinks [50,51,52] and further selected variables that directly reflect the carbon sink by terrestrial vegetation in China, and variables that indirectly reflect China’s carbon sinks, namely “Forest carbon sink costs, Forest carbon sink efficiency, Ocean carbon sink efficiency, and Ocean carbon sink potential”. The results are shown in the table below.

The results in Table 6 indicate that the CTPP can significantly reduce the forest carbon sink cost and significantly improve forest carbon sink efficiency, ocean carbon sink efficiency, and ocean carbon sink potential. This may be due to the fact that the CTPP can control pollution from the source by improving urban governance, such as reducing emissions of sewage and waste gases, increasing the recycling rate of municipal and industrial waste, controlling the flow of urban sewage into rivers and seas, and controlling the flow of smoke and waste gases into forests. These measures to control pollution sources further protect the carbon sink capacity of forests and oceans and reduce the carbon sink costs of forests. Furthermore, no matter what kind of carbon sink, it will be included in the CTM as a carbon sink project, and the trading of carbon sink projects in the CTM will lead to an increase in carbon sinks and economic development. For example, the government of coastal areas in China has the right to manage marine resources. As the owner and trader of carbon sinks, the more the local government cares about the environment, the more active it will be to participate in CTM’s ocean carbon sink project. The government will then increase environmental protection policies and measures to obtain more ocean carbon sink resources and the amount of ocean carbon sink to trade in the CTM. Eventually, the proceeds will be ploughed back into the protection of ocean carbon sinks. In this way, carbon sinks are increased, pollution is controlled, and the economy is developed. In summary, the results showed that the impact of the CTPP on carbon reduction and carbon sink in China is significant and effective. This means that China’s CTM can further contribute to achieving the carbon neutrality target.

### 5.3. Mechanism Analysis

Based on the above theoretical analysis, it can be seen that the impact of the CTPP on the net carbon sink may be realized through three mechanisms: the effect on concern for the environment, the effect on urban governance, and the effect on energy production and consumption. Therefore, Hypotheses 2 and 4 are tested in this paper. Firstly, for the effect on concern for the environment, the frequency of words related to the term “environmental protection” (Environment-related terms include environmental protection, environmental protection, pollution, energy consumption, emission reduction, emissions, ecology, green, low-carbon, air, chemical oxygen demand, sulfur dioxide, carbon dioxide, PM_10_, and PM_2.5_) in local government work reports as a proxy variable for ecological governance is measured by the existing study [49]. On the one hand, more words related to “environmental protection” could reflect the government’s concern for the environment. On the other hand, local government work reports usually occur at the beginning of the year. In contrast, economic activities occur throughout the year; they can also alleviate endogenous problems to a certain extent. Secondly, for the effect on urban governance, this paper adopts the urban household waste harmless treatment rate and industrial solid waste comprehensive utilization rate to express this effect. Finally, for the effect on energy production and consumption, this paper uses urban hydroelectric power generation, coal consumption structure share, and energy consumption intensity to express this effect. Referring to the methods used in existing studies to analyze mechanisms [53,54,55], this paper constructs the following models:

(3) $$M_{it}=\beta_{0}+\beta_{1}DID_{it}+\beta_{2}\;\mathrm{contro}l_{it}+cs_{i}+year_{t}+qy_{rt}+\varepsilon_{it}$$

where *M_it_* is the mechanism variable group consisting of the effect on concern for the environment, the effect on urban governance, and the effect on energy production and consumption, and precisely the frequency of words expressing government concern for the environment, the urban household waste harmless treatment rate and the industrial solid waste comprehensive utilization rate, urban hydroelectric power generation, coal consumption structure share, and energy consumption intensity. The meanings of the remaining variables are shown in Equation (1). The specific tests are shown in Table 7.

The results in Table 7 indicate that the regression coefficients of DID variables are significantly positive for the effect of government environmental concern, indicating that the CTPP can further promote increasing the net carbon sink by increasing the government’s concern for the environment. Regarding the effect on urban governance, the regression coefficients of DID variables are significantly positive, indicating that the CTPP can further promote increasing the net carbon sink by increasing the rate of harmless treatment of urban domestic waste and the comprehensive utilization rate of industrial waste. Regarding the effect on energy production and consumption, the regression coefficients of DID variables are significantly positive for hydroelectric power generation and negative for coal consumption structure and energy consumption intensity, indicating that the CTM can further promote increasing the net carbon sink by increasing the production of new energy and reducing the consumption of high-carbon energy and energy intensity, and finally, accelerating the achievement of the “carbon neutrality” goal. Therefore, hypotheses 2–4 are proved.

### 5.4. Analysis of the Moderating Effect

While the impact of the CTPP on regional net carbon sink can be realized through the three mechanisms: the effect on concern for the environment, the effect on urban governance, and the effect on energy production and consumption, it may also be moderated by related factors. Therefore, this paper analyzes the moderating role of the relevant variables on the impact of the CTPP on regional net carbon sinks from the perspective of both enterprises and markets. First, on the enterprise side, the impact of the CTM on the net carbon sink is reflected in enterprises’ profit and production cost. On the one hand, one of the main reasons for implementing the CTPP is to restrain enterprises’ negative environmental externality behavior. According to behavioral psychology theory, any decision of an individual is determined by his or her will. If an enterprise is willing to manage its environment, it will have a better positive environmental externality behavior, so it will be more active in environmental management, have more net carbon quotas, and will actively participate in CTM transactions to increase its profits. Therefore, this paper argues that enterprises’ willingness to engage in environmental management can positively regulate the impact of the CTPP on regional net carbon sinks; on the other hand, the carbon quota system increases the production cost of enterprises to a certain extent [2], which poses a severe challenge to the market competitiveness of enterprises [1]. It forces enterprises to improve their total factor productivity and even squeezes out low-productivity enterprises from the market through the process whereby “-The best wins, the worst [are] eliminated” [2], thus promoting the effectiveness of the CTM. Therefore, this paper argues that the higher the total factor productivity of enterprises, the more likely it is to positively regulate the impact of the CTPP on regional net carbon sinks. Second, in terms of the internal carbon market, it is easy to see that the more effective the core operating mechanism of the carbon market, the more liquid the carbon market, the higher the price of the carbon market, the larger the carbon market and the stronger the penalties, the better the construction and development of the carbon market can be improved and promoted [2], thus further positively regulating the impact of the carbon market on regional net carbon sinks. In summary, in regard to corporate willingness to engage in environmental governance, this paper uses whether the company has a vision or values related to environmental responsibility and whether the company is willing to care about the environment to express corporate willingness to engage in environmental governance. If both areas of willingness exist, it is recorded as 2. If only one area of willingness exists, it is recorded as 1, and if neither area of willingness exist, it is recorded as 0. In regard to corporate total factor productivity, this paper refers to the study of Yang (2015) [56], and use the LP and OP methods to calculate the total factor productivity of the company; for the internal factors of the CTM, this paper refers to the study of Wu et al. (2021) [2], which uses the logarithm of the annual average daily closing price to represent the CTM price, the logarithm of the annual number of non-zero trading days to represent the CTM liquidity, and the ratio of the annual cumulative trading size of each region’s CTM to the region’s annual carbon emissions to represent the CTM size. In addition, this paper also refers to the study of Liu et al. (2021) [57] to classify the penalty intensity of the CTM in each pilot region and records Beijing, Shanghai, and Shenzhen as 6, Chongqing as 5, Hubei as 4, Guangdong as 3, Fujian as 2, Tianjin as 1, and the rest as 0. The test results of each specific moderating effect are shown in Table 8.

As the results in Table 8 indicate, the interaction terms of DID variables with corporate environmental management intention, corporate total factor productivity, carbon market price, carbon market liquidity, carbon market size, and carbon market penalty are all significantly greater than 0. This indicates that corporate environmental management intention, corporate total factor productivity, carbon market price, carbon market liquidity, carbon market size, and carbon market penalty can positively regulate the effect of the CTM on regional net carbon sinks.

### 5.5. Heterogeneity Analysis

Considering that intergroup differences in relevant factors can lead to differences in the effects of the CTPP on regional net carbon sinks, this paper explores the heterogeneity in three aspects: regional technology endowment, differences in pilot regions, and regional state-owned assets share. The reasons for this are threefold: Firstly, technology is the first productive force that drives the economy, and the economy is closely related to the environment, so the difference in technology endowment between regions will inevitably have a heterogeneous effect on the net carbon sink effect of the CTM. Secondly, the difference in the degree of economic development of each pilot region will also have a heterogeneous effect on the net carbon sink effect of the CTM. Finally, the proportion of state-owned enterprises in the region, to a certain extent, reflects the regional differences in enterprise dynamics. The carbon quotas in the CTM will impact the profits and costs of enterprises. They will inevitably also show heterogeneous effects on the net carbon sink effect with the difference in the number of the enterprises‘ nature between regions. Therefore, in this paper, the total number of invention patents in each region is differentiated according to the mean value, and regions are thereby divided into high-technology endowment and low-technology endowment regions, for heterogeneous exploration. Secondly, this paper also divides the CTPP regions into two samples—Beijing, Shanghai, and Guangdong; Tianjin, Chongqing, and Hubei—according to their economic levels for heterogeneity exploration. Finally, this paper also divides the share of fixed assets of state-owned enterprises in industrial enterprises above the scale in each region by median and divides them into a low share of state-owned enterprises and a high share of state-owned enterprises for heterogeneity exploration. The specific test results are shown in Table 9.

The results in Table 9 indicate that the impact of the CTPP on regional net carbon sinks is more significant in high-technology-endowed regions as compared to low-technology-endowed regions, which is consistent with the findings of existing studies [2]. A possible reason for this is that regions with higher technological endowments will see more green invention innovations emerge, thus increasing the net carbon sink effect. Secondly, the impact of the CTPP on Beijing, Shanghai, and Guangdong is more significant than that in Tianjin, Chongqing, and Hubei. A possible reason for this is that the development of the CTM and the intensity of administrative interventions are relatively higher in Beijing, Shanghai, and Guangdong, which leads to a higher net carbon sink effect. Finally, the impact of CTPP on regional net carbon sinks is more significant in regions with a low share of state-owned assets, as compared to regions with a high share of state-owned assets. A possible reason for this is that carbon quotas in the CTM impact the profits and costs of enterprises, and non-state enterprises are more sensitive to changes in profits and costs in the face of fierce market competition, so non-state enterprises will focus more on improving the quality of green innovation to gain a competitive advantage in the carbon CTPP [58], which further increases the net carbon sink of the region.

## 6. Deficiencies and Prospects

This paper has some limitations. First, as mentioned above, under ideal conditions, the carbon sequestration of more plants, soils, and oceans should be measured and summed up in a uniform way to obtain further data reflecting the complete natural carbon sink of nature. However, as carbon sink data for water bodies, such as oceans and lakes, are difficult to estimate accurately and current human activities through land use change mainly affect terrestrial vegetation, this paper is subject to constraints regarding the completeness, accessibility, scientific validity, and uniformity of the current data measurement. This paper can only select and use the most complete and scientific carbon sequestration data available for terrestrial vegetation and then match it with carbon emissions data of the same measurement caliber and accuracy to obtain data with low measurement error. Therefore, future studies can further explore more comprehensive carbon sequestration data to ensure the further development of the study. Second, this paper mainly focuses on the CTPP, but in the real market, policies do not work independently of each other and often need to be complemented by other policies. Therefore, future research can further explore the impact and mechanism of environment-related policies on “double carbon” and compare them with the CTPP to identify similarities, differences, and interactions between them. Thirdly, this paper explores the CTPP only at the macro level of policy and does not go into the internal structure of the CTPP. For example, carbon emissions allowances and nationally certified voluntary emission reductions are two basic trading products that have emerged in the trading market. Whether these underlying traded products have a different impact on “carbon neutrality“ is worth investigating in depth. Some studies have already started to explore the impact of the quota approach on the competitiveness of high-polluting enterprises in terms of Carbon Quota Benchmark Allocation [59]. The findings of these studies are insightful, so future research could look at the internal structure of the CTPP and explore the impact of different carbon quota approaches and carbon trading products on China‘s “double carbon” targets so that more specific and targeted conclusions and recommendations can be drawn. Finally, the reality is that the implementation of the CTPP may be regulated and influenced at a more micro level, in addition to the interaction and influence of other macro policies. For example, some current studies have found that total urban carbon emissions are closely related to surface temperature [60]. The results of the study are of great reference value to policy makers in formulating policies to reduce emissions and achieve sustainable urban development. As a large country, China certainly has some heterogeneity in surface temperature, and it is worthwhile to examine whether this heterogeneity further increases or decreases the impact of the CTPP on the achievement of the “double carbon” target. In addition, China’s CTM is also actively exploring personal carbon trading, and no studies have yet been conducted to explore whether personal carbon trading can further contribute to the achievement of the “double carbon” goal. Only some cutting-edge research has explored the willingness of the urban public to pay for heat mitigation and adaptation in terms of the greenhouse effect caused by CO_2_ [61]. These studies have set the direction for future exploration so that further comprehensive and systematic studies on the CTPP can be conducted from a more microscopic and specific perspective in the future, thus providing more diverse experiences and ideas on carbon pollution in China and the world. In addition, the research conclusions of this paper only focus on the impact of the CTM on net carbon sinks at the Chinese level and do not extend to the global level. In contrast, foreign countries have established the CTM earlier than China and have more theoretical research and practical experience than China. For example, early research on terrestrial carbon sinks has shown that terrestrial carbon sinks in the US are one of the most cost-effective options for mitigating greenhouse gas emissions [62]. The CTM can be used to promote carbon sequestration in terrestrial forests, among which the more important measures are to ensure the long-term nature of carbon emission reduction, reduce the leakage caused by forest land conversion, and establish a national carbon credit system [63]. These studies have not yet been conducted in China. Therefore, compared to foreign research, the CTM research in China is still in its early stages. As China has the largest CTM in the world, the impact of China’s CTM on net carbon sinks can be further explored in the future. For example, how China’s CTM can achieve long-term net carbon sinks, reduce leakage of net carbon sinks, and establish carbon credits can be explored. This will contribute to not only the achievement of China’s “carbon neutrality” target but also the healthy operation of the global carbon market.

## 7. Conclusions and Policy Implications

### 7.1. Conclusions

This paper’s assessment of the impact of the CTPP on achievement of the “carbon neutrality” target is a valuable addition to existing studies and an essential basis for developing a comprehensive CTM in China. Based on a panel of 283 Chinese cities in the period from 2006 to 2017, this paper examines the impact of the CTPP on achieving the “carbon neutrality” target. The study shows that the CTPP can promote an increase in regional net carbon sinks and accelerate the achievement of the “carbon neutrality” target. The findings are validated by a series of robustness tests, including a parallel trend test, a placebo test, and PSM-DID. The mechanism analysis reveals that the carbon-neutral goal of the CTPP can be achieved through the effect on concern for the environment, the effect on urban governance, and the effect on energy production and consumption. Specifically, the CTPP can further promote increasing the net carbon sink by increasing the government’s concern for the environment, increasing the rate of harmless urban domestic waste treatment, increasing the total utilization rate of industrial solid waste, increasing the amount of hydroelectric power generation and reducing the consumption of high-carbon energy and energy consumption intensity. Further analysis reveals that enterprises’ willingness and production behavior, as well as the internal factors of the market, have a moderating effect on the achievement of the “carbon neutrality” goal in the CTPP. Specifically, enterprises’ willingness to manage the environment, total factor productivity, and the price, liquidity, market size, and penalty of the carbon market play a positive moderating role in promoting the increase of regional net carbon sinks in the CTPP. In addition, there is heterogeneity among regions with different technological endowments, CTPP regions, and regions with state-owned assets regarding the impact of the CTPP on achieving the “carbon neutrality” goal. Specifically, the impact of the CTPP on regional net carbon sinks is more significant in regions with high technology endowment than in regions with low technology endowment; the impact of the CTPP on regional net carbon sinks is more significant in Beijing, Shanghai and Guangdong than in Tianjin, Chongqing, and Hubei; and the impact of the CTPP on regional net carbon sinks is more significant in regions with a low share of state-owned assets than those with a high share of state-owned assets. This paper provides critical practical references and empirical evidence that can help China to better achieve the “double carbon” goal.

### 7.2. Policy Implications

Based on the above findings, this paper proposes the following policy recommendations: (1) This paper shows that the CTPP has a positive effect on the net carbon sink of a region. Therefore, in the process of accelerating the development of the CTM, the Chinese government should not only use and protect the carbon sequestration capacity of nature but also shift the focus of the policy design with respect to “carbon emission reduction” by introducing a series of market policies to “increase carbon sequestration”, to achieve a significant increase in net carbon sinks and steady economic growth. (2) The research in this paper shows that the CTPP can further contribute to increasing the net carbon sink through the effect on concern for the environment, the effect on urban governance, and the effect on energy production and consumption. Therefore, the Chinese government should actively promote environmental protection and ensure the right to engage in actions related to environmental governance so that the public can actively participate in environmental and urban governance while increasing their levels of environmental knowledge and concern. In this way, not only can the government share the pressure of environmental management, but the public can also realize that cities’ environmental management needs to be maintained by all sectors of society. In addition, the Chinese government should encourage enterprises to produce new energy and reduce high-energy consumption through subsidies and taxation mechanisms to promote and force enterprises to achieve “clean” energy production and consumption. (3) This paper shows that the willingness of enterprises and the internal elements of the market positively influence the achievement of the carbon-neutral goal of the CTPP. Therefore, enterprises themselves should be aware of the importance of environmental protection and should actively take up environmental and social responsibility from the inside out. In regard to the CTM, the government should also play an active role as the “visible hand” and intervene in the internal factors of the CTM to stabilize and expand the CTM. (4) This paper shows that there is heterogeneity among regions with different technology endowments, CTPP regions, and regions with state-owned assets, which influences the achievement of the “carbon neutrality” goal in the CTPP. Therefore, the Chinese government should design differentiated market policies based on regional heterogeneity: for example, for regions with higher technology endowment, it should increase environmental penalties and creative innovation subsidies to force and promote inventive green innovation in the region. For regions with lower technology endowments, the cost of financing constraints should be moderately alleviated, and innovative subsidies should be increased to ensure an increase in regional green innovation output in the first place.

## Figures and Tables

**Table 1:** Descriptive statistics.

Variable	Obs	Mean	Std. Dev.	Min	Max
Ncs	3396	1.26	40.157	−215.447	442.56
Rgdp	3396	0.987	0.712	−1.289	3.072
Rgdp^2^	3396	1.48	1.582	0	9.439
Is	3396	0.378	0.093	0.086	0.806
Es	3379	0.357	0.102	0	0.826
Dotow	3222	29.419	28.539	0.02	311.13
Pd	3382	430.61	331.529	4.7	2648.11
Mi	3396	6.66	1.622	2.372	11.109
Nie	3395	6.485	1.119	0	7.507
Dfd	3381	0.071	0.029	0.019	0.238
Dupie	3315	1.007	0.687	0.052	17.141

**Table 2:** Baseline regression.

	(1)	(2)	(3)	(4)	(5)	(6)
	Ncs	Ncs	Ncs	Ncs	Ncs	Ncs
*DID*	2.508 **	12.180 ***	7.885 *	3.049 ***	2.451 **	2.713 **
	(0.999)	(3.957)	(4.055)	(1.049)	(1.064)	(1.092)
*Rgdp*		−11.166 **	−18.359 ***	−2.894 ***	7.540 *	6.607
		(5.366)	(6.189)	(0.961)	(4.543)	(4.886)
*Rgdp2*		−3.622 *	−2.767	−3.108 ***	−2.899 ***	−3.067 ***
		(1.937)	(2.021)	(0.603)	(0.627)	(0.767)
*Is*		10.511	24.349	2.176	−6.442	−3.555
		(20.475)	(20.367)	(5.965)	(7.855)	(8.127)
*Es*		−9.541	−33.037 **	6.089 *	6.642	5.583
		(14.453)	(15.661)	(3.269)	(4.075)	(4.148)
*Dotow*		−0.065	−0.011	0.016 *	0.010	0.004
		(0.053)	(0.055)	(0.009)	(0.009)	(0.011)
*Pd*		−0.065 ***	−0.064 ***	−0.004 *	−0.001	−0.001
		(0.008)	(0.008)	(0.002)	(0.002)	(0.002)
*Mi*		2.703 ***	3.503 ***	1.098 ***	0.010	−0.234
		(0.959)	(1.191)	(0.124)	(0.377)	(0.460)
*Nie*		0.009	0.465	0.179 *	0.205 **	0.200 **
		(0.730)	(0.714)	(0.100)	(0.092)	(0.091)
*Dfd*		−83.528	−145.750 **	−29.727 **	−18.369	−22.936 *
		(61.117)	(60.890)	(12.770)	(12.035)	(13.344)
*Dupie*		1.970	0.606	0.301 **	0.217	0.211
		(1.505)	(1.446)	(0.144)	(0.168)	(0.171)
*Constant*	1.120 ***	32.372 ***	36.993 ***	−0.492	−2.609	−0.043
	(0.056)	(8.101)	(9.194)	(2.123)	(7.127)	(7.618)
time-fixed	Y		Y		Y	Y
city-fixed	Y			Y	Y	Y
region-year fixed	Y					Y
Observations	3396	3132	3132	3132	3132	3132
R-squared	0.989	0.421	0.436	0.988	0.990	0.990

Note: *** *p* < 0.01, ** *p* < 0.05, * *p* < 0.1; In brackets are clustering standard errors at the city level; The notes and descriptions of all the tables below are consistent with Table 2.

**Table 3:** Parallel trend test.

	(1)
	Ncs
*pre5*	−0.521
	(0.682)
*pre4*	−0.205
	(1.030)
*pre3*	−0.287
	(1.399)
*pre2*	0.635
	(1.465)
*pre1*	0.301
	(1.336)
*current*	1.939
	(1.544)
*post1*	2.462 *
	(1.447)
*post2*	2.974 *
	(1.630)
*post3*	3.172 *
	(1.686)
*post4*	5.244 **
	(2.322)
*Rgdp*	6.701
	(4.862)
*Rgdp^2^*	−3.051 ***
	(0.773)
*Is*	−3.642
	(8.292)
*Es*	5.413
	(4.129)
*Dotow*	0.005
	(0.011)
*Pd*	−0.001
	(0.002)
*Mi*	−0.379
	(0.476)
*Nie*	0.201 **
	(0.092)
*Dfd*	−23.538 *
	(13.300)
*Dupie*	0.243
	(0.181)
*Constant*	0.988
	(7.715)
time-fixed	Y
city-fixed	Y
region-year fixed	Y
Observations	3132
R-squared	0.990

**Table 4:** Other robustness tests: PSM-DID and excluding the effects of other relevant policies in the same period.

	(1)	(2)	(3)	(4)
	Nearest Neighbor Matching	Radius Matching	Exclusion Low Carbon Pilot	Exclusion Atmospheric Pollution Key Control Area
	Ncs	Ncs	Ncs	Ncs
*DID*	3.771 **	3.713 **	3.203 **	5.715 **
	(1.488)	(1.491)	(1.383)	(2.306)
*Rgdp*	−0.333	−0.595	8.970	−5.429
	(9.125)	(9.152)	(9.582)	(19.971)
*Rgdp^2^*	−3.288 ***	−3.284 ***	−3.094 ***	−2.233
	(0.917)	(0.941)	(0.763)	(3.097)
*Is*	−28.643 *	−29.032 *	−8.783	5.373
	(14.912)	(15.062)	(15.466)	(32.791)
*Es*	15.500	14.950	3.893	16.779
	(12.516)	(12.559)	(6.396)	(38.285)
*Dotow*	0.009	0.009	0.006	−0.006
	(0.016)	(0.016)	(0.017)	(0.032)
*Pd*	0.0002	0.0001	0.001	0.003
	(0.002)	(0.002)	(0.003)	(0.004)
*Mi*	−1.773 **	−1.673 *	−0.875	−0.949
	(0.853)	(0.943)	(0.784)	(1.503)
*Nie*	0.158	0.163	0.289 *	0.673
	(0.142)	(0.142)	(0.152)	(0.495)
*Dfd*	−0.763	−3.768	−10.016	−10.233
	(33.324)	(34.117)	(28.558)	(59.214)
*Dupie*	0.067	0.079	0.427	−0.001
	(0.178)	(0.181)	(0.379)	(0.516)
*Constant*	18.284	18.818	0.582	−26.592
	(12.054)	(12.216)	(16.178)	(49.288)
time-fixed	Y	Y	Y	Y
city-fixed	Y	Y	Y	Y
region-year fixed	Y	Y	Y	Y
Observations	1189	1153	1382	545
R-squared	0.574	0.562	0.991	0.982

**Table 5:** Other robustness tests: excluding the effect of special samples and replacing the *dependent* variables.

	(1)	(2)	(3)	(4)	(5)
	Exclude All Municipalities Directly under the Central Government Ncs	Exclude Chongqing Ncs	Exclude Fujian and Sichuan Ncs	Ln Carbon Sequestration−Ln carbon Emission Ncs	PM_2.5_
*DID*	1.994 ***	2.713 **	3.102 **	0.066 ***	−0.028 **
	(0.611)	(1.128)	(1.247)	(0.018)	(0.012)
*Rgdp*	10.139 ***	6.454	4.839	−0.122	−0.000
	(3.738)	(4.877)	(4.999)	(0.105)	(0.055)
*Rgdp^2^*	−2.907 ***	−3.035 ***	−2.894 ***	0.013	0.010
	(0.762)	(0.772)	(0.780)	(0.009)	(0.006)
*Is*	−1.137	−3.946	−3.395	0.079	0.094
	(7.439)	(8.227)	(9.517)	(0.125)	(0.108)
*Es*	6.405 **	5.119	3.437	0.250 **	−0.036
	(3.167)	(4.112)	(4.282)	(0.100)	(0.060)
*Dotow*	0.006	0.008	0.008	0.001 **	0.000
	(0.009)	(0.011)	(0.011)	(0.000)	(0.000)
*Pd*	−0.001	−0.001	−0.003	0.000	−0.000
	(0.002)	(0.002)	(0.002)	(0.000)	(0.000)
*Mi*	0.029	−0.201	−0.038	−0.017 **	−0.002
	(0.363)	(0.464)	(0.504)	(0.008)	(0.008)
*Nie*	0.179 **	0.201 **	0.217 **	0.002	−0.000
	(0.086)	(0.091)	(0.100)	(0.002)	(0.002)
*Dfd*	−25.195 **	−20.787	−25.596 *	−0.481 *	0.243
	(11.826)	(13.149)	(13.769)	(0.269)	(0.189)
*Dupie*	0.138	0.199	0.313	0.003	0.001
	(0.154)	(0.167)	(0.255)	(0.004)	(0.005)
*Constant*	−5.113	−0.098	0.306	0.041	3.504 ***
	(6.306)	(7.682)	(8.248)	(0.154)	(0.101)
time-fixed	Y	Y	Y	Y	Y
city-fixed	Y	Y	Y	Y	Y
region-year fixed	Y	Y	Y	Y	Y
Observations	3084	3120	2814	3132	3132
R-squared	0.990	0.990	0.990	0.995	0.967

**Table 6:** Carbon emissions, carbon uptakes, and other carbon sinks.

	(1)	(2)	(3)	(4)	(5)	(6)
	Carbon Emissions	Carbon Sink	Forest Carbon Sink Costs	Forest Carbon Sink Efficiency	Ocean Carbon Sink Efficiency	Ocean Carbon Sink Potential
*DID*	−2.527 **	0.185	−0.169 ***	0.245 ***	0.155 ***	0.187 ***
	(1.133)	(0.413)	(0.062)	(0.036)	(0.040)	(0.030)
*Rgdp*	−8.009 *	−1.402	0.546 *	−0.114	−0.289	0.816 ***
	(4.508)	(1.224)	(0.302)	(0.115)	(0.180)	(0.192)
*Rgdp^2^*	3.038 ***	−0.029	0.097 **	−0.004	−0.042	0.054 **
	(0.890)	(0.328)	(0.044)	(0.016)	(0.028)	(0.025)
*Is*	−1.798	−5.354	−0.666	−0.451 **	−1.378 ***	1.425 ***
	(7.391)	(6.033)	(0.578)	(0.228)	(0.347)	(0.317)
*Es*	−5.693	−0.110	−0.600	−0.317 *	−0.325	−0.137
	(3.944)	(1.502)	(0.513)	(0.167)	(0.227)	(0.214)
*Dotow*	−0.002	0.002	0.003 ***	−0.001 *	−0.000	−0.000
	(0.011)	(0.003)	(0.001)	(0.000)	(0.000)	(0.001)
*Pd*	0.001	−0.000	−0.000	−0.000	−0.000	−0.000
	(0.002)	(0.000)	(0.000)	(0.000)	(0.000)	(0.000)
*Mi*	0.321	0.087	0.120 ***	0.072 ***	0.584 ***	−0.014
	(0.492)	(0.251)	(0.033)	(0.018)	(0.038)	(0.034)
*Nie*	−0.228 **	−0.028	0.021	−0.003	0.001	−0.004
	(0.092)	(0.045)	(0.018)	(0.004)	(0.006)	(0.006)
*Dfd*	11.323	−11.614	0.422	1.173 ***	0.192	−0.465
	(12.435)	(7.956)	(1.265)	(0.450)	(0.746)	(0.733)
*Dupie*	−0.263	−0.051	0.017	0.001	−0.013	−0.019
	(0.178)	(0.084)	(0.019)	(0.010)	(0.012)	(0.014)
*Constant*	32.692 ***	32.649 ***	4.430 ***	0.313	−2.088 ***	−1.085 **
	(7.289)	(3.576)	(0.583)	(0.238)	(0.471)	(0.491)
time−fixed	Y	Y	Y	Y	Y	Y
city−fixed	Y	Y	Y	Y	Y	Y
region−year fixed	Y	Y	Y	Y	Y	Y
Observations	3132	3132	752	3132	212	212
R−squared	0.981	0.992	0.895	0.691	0.966	0.945

**Table 7:** Mechanism analysis.

	(1)	(2)	(3)	(4)	(5)	(6)
	Government Environmental Concern	Industrial Solids Utilization Rate	Hazard-Free Garbage Treatment Rate	Hydroelectricity	Coal Consumption Structure	Energy Consumption Intensity
*DID*	0.0004 *	4.508 *	5.510 **	158.492 ***	−0.079 ***	−0.063 ***
	(0.000)	(2.498)	(2.361)	(31.246)	(0.016)	(0.010)
*Rgdp*	0.002 ***	−6.931	−14.887	630.498 ***	−0.245 **	−0.615 ***
	(0.001)	(7.857)	(11.848)	(172.750)	(0.096)	(0.059)
*Rgdp^2^*	−0.000 ***	0.519	−8.327 ***	−76.129 ***	0.017	−0.020 ***
	(0.000)	(1.491)	(1.525)	(29.344)	(0.011)	(0.006)
*Is*	0.001	−28.750 *	−33.356	−181.252	−0.132	−0.055
	(0.001)	(17.378)	(26.652)	(288.457)	(0.131)	(0.081)
*Es*	−0.001 *	−1.145	19.865	497.288 **	−0.074	0.063
	(0.001)	(9.905)	(14.116)	(199.701)	(0.079)	(0.055)
*Dotow*	0.000	−0.016	−0.011	−0.411 *	−0.000	−0.000 ***
	(0.000)	(0.022)	(0.028)	(0.216)	(0.000)	(0.000)
*Pd*	−0.000	0.002	−0.011 **	0.028	−0.000	−0.000 ***
	(0.000)	(0.003)	(0.005)	(0.042)	(0.000)	(0.000)
*Mi*	−0.000	0.142	−0.351	−155.200 ***	0.048 ***	0.033 ***
	(0.000)	(1.334)	(1.318)	(29.777)	(0.015)	(0.005)
*Nie*	−0.000	0.023	−0.326	−1.243	−0.000	−0.002 *
	(0.000)	(0.281)	(0.417)	(3.317)	(0.002)	(0.001)
*Dfd*	0.002	42.459	−73.415 *	407.869	−0.403	0.182
	(0.002)	(35.652)	(43.082)	(542.033)	(0.293)	(0.155)
*Dupie*	0.000	−0.817	0.651	11.180	−0.002	−0.003
	(0.000)	(0.648)	(0.765)	(11.760)	(0.003)	(0.003)
*Constant*	0.003 **	75.854 ***	132.600 ***	730.567 **	1.009 ***	−6.040 ***
	(0.001)	(14.954)	(21.950)	(291.148)	(0.177)	(0.081)
time-fixed	Y	Y	Y	Y	Y	Y
city-fixed	Y	Y	Y	Y	Y	Y
region-year fixed	Y	Y	Y	Y	Y	Y
Observations	3052	3034	2930	3115	3132	3132
R-squared	0.481	0.701	0.507	0.910	0.949	0.997

**Table 8:** Analysis of regulation effects.

	(1)	(2)	(3)	(4)	(5)	(6)	(7)
	Ncs	Ncs	Ncs	Ncs	Ncs	Ncs	Ncs
DID * Environmental Governance Willingness	0.266 * (0.141)						
DID * Productivity _op		0.243 ***					
		(0.037)					
DID * Productivity _lp			0.241 ***				
			(0.037)				
DID * Carbon Market Liquidity				0.417 **			
				(0.210)			
DID * Carbon Market Price					1.685 ***		
					(0.594)		
DID * Carbon Market Size						37.622 ***	
						(14.048)	
DID * Carbon Market Penalties							4.879 ***
							(1.203)
time-fixed	Y	Y	Y	Y	Y	Y	Y
city-fixed	Y	Y	Y	Y	Y	Y	Y
region-year fixed	Y	Y	Y	Y	Y	Y	Y
Observations	18064	27817	27817	3132	3132	3132	3132
R-squared	0.994	0.992	0.992	0.990	0.990	0.990	0.991

Note: Given the length of the table, the results of the moderating effects are reported with the core explanatory variables, moderating variables, and control variables omitted.

**Table 9:** Heterogeneity analysis.

	(1)	(2)	(3)	(4)	(5)	(6)
	High Technology Endowment Ncs	Low Technology Endowment Ncs	Beijing, Shanghai and Guangdong Ncs	Tianjin, Chongqing and Hubei Ncs	Low—Share of State-Owned Assets Ncs	High—Share of State-Owned Assets Ncs
*DID*	3.238 **	0.045	2.254 **	0.982 *	1.762 ***	5.572
	(1.308)	(0.687)	(0.992)	(0.497)	(0.645)	(3.384)
time-fixed	Y	Y	Y	Y	Y	Y
city-fixed	Y	Y	Y	Y	Y	Y
region-year fixed	Y	Y	Y	Y	Y	Y
Observations	1535	1583	363	258	1520	1541
R-squared	0.991	0.991	0.995	0.995	0.993	0.991

Note: Given the length of the table, the results of the heterogeneity analysis are reported with the control variables omitted.

## Data Availability

The data used to support the findings of this study are available from the corresponding author upon request.
